# One-Step Cannulation and Distance Measurement during Aortic Branched Endograft Repair: The Neuron Catheter Trick

**DOI:** 10.3390/medsci12040054

**Published:** 2024-10-06

**Authors:** Gioele Simonte, Gianluigi Fino, Gianbattista Parlani, Rachele Simonte, Giacomo Isernia

**Affiliations:** 1 Vascular and Endovascular Surgery Unit, S. Maria Della Misericordia University Hospital, 06132 Perugia, Italy; gianluigi.finomd@gmail.com (G.F.); parlani.gianbattista@gmail.com (G.P.); iserniagiacomo1@gmail.com (G.I.); 2 Department of Medicine and Surgery, Università Degli Studi di Perugia, 06123 Perugia, Italy; rachele.simonte@gmail.com

**Keywords:** complex aortic aneurysm, BEVAR, FEVAR, branched endograft, catheter, bridging stent

## Abstract

Purpose: This paper aims to describe a straightforward, efficient, and reliable technique to simplify cannulation maneuvers during aortic branched endograft repair. Technique: The suggested approach utilizes the Penumbra Neuron Select catheter, which combines diagnostic, sizing, and support capabilities in one. This has the potential to reduce procedural time and minimize the need for serial catheter and guidewire exchanges. Conclusions: The proposed technique offers a simple yet effective tool to mitigate the risk of vessel loss and injury, and to streamline complex aortic repair procedures.

## 1. Introduction

Catheterization of visceral vessels and precise deployment of bridging stents remain challenging steps in complex aortic procedures [1]. The techniques for both inner and outer branched endograft catheterization are continuously evolving and vascular surgeons are adopting innovative strategies to address complex situations [2,3]. This note presents a pragmatic and easily applicable maneuver utilizing the Neuron Penumbra Select catheter (Penumbra Inc., Alameda, CA, USA) to simplify the process from branch cannulation to bridging component release. This approach has the potential to reduce procedural time and the risk of target vessel loss during catheter replacement with sizing devices, required to measure the length to be covered and select the appropriate bridging component. Institutional review board approval was not necessary for this report.

### 1.1. Device Features

The Neuron catheter family, initially designed for neurovascular applications, has broad applications across vascular domains [4,5]. It combines the features of a diagnostic hydrophilic catheter, a sizing catheter, and a crossing catheter. Its design allows for the engagement of tortuous anatomy and atraumatic vessel access due to its stainless steel braided proximal shaft for support and torque response of the soft polymer distal tip. The seamless transition zone facilitates smooth advancement. Available in 5 F (120–130 cm) and 6 F (105/125 cm) configurations, both with a 0.040-inch inner luminal diameter for diagnostic angiography, they are compatible with 0.035″–0.038″ (0.89 mm–0.97 mm) guidewires.

**Figure 1:**
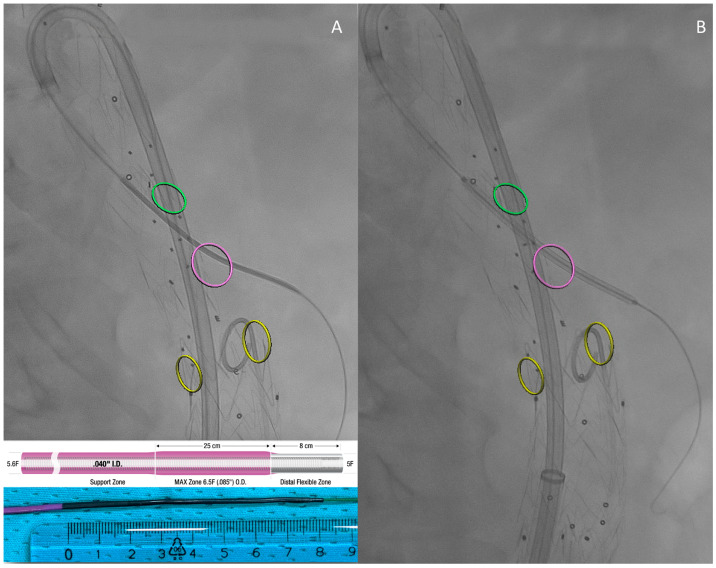
Intraoperative images: Once the target vessel has been cannulated, the characteristic 8 cm-long radiopaque Neuron tip, aligned with the branch inlet, allows for measurement of the gap to be covered (**A**). The bridging component is then advanced through the branch and the target vessel, whose origin is marked in this case with a fusion marker (a purple circle) (**B**). Bottom left: Neuron catheter tip detail. Circles mark the origin of reno–visceral vessels according to fusion guidance: green is for the Celiac Trunk, purple for the Superior Mesenteric Artery (the vessel about to be bridged), and yellow for the renal arteries.

The 6 F version has a 5.6 F coil reinforced shaft with a double transition zone in its distal 33 cm: the proximal 25 cm has an outer diameter of 0.085″ (6.5 F) tapering to 5 F, with a flexible, radiopaque 8 cm distal zone (Figure 1). Four tip configurations are available: SIM-V, BERN, H1, and SIM [4].

### 1.2. Technique

Catheterization of visceral vessels and stable introducer sheath positioning are pivotal in complex aortic procedures. Proper orientation, precise endograft release, and ostial stenosis avoidance are crucial in preventing vessel loss and procedural failure [1,6].

The standard catheterization approach involves advancing a 0.035″ hydrophilic guidewire with a 5 Fr angulated diagnostic catheter [7].

Sequential cannulation maneuvers often necessitate various techniques, such as anchoring or the use of steerable introducer sheaths [3,8].

Following vessel catheterization, standardized steps include selective angiography, guiding wire positioning, and measuring the length to be covered by the bridging component, followed by introducer sheath or stent-graft placement.

This typically involves replacing the diagnostic catheter with a sizing or balloon catheter.

The proposed technique can be used after standard or partial deployment of branched thoracoabdominal aortic endografts [9], for both antegrade (axillary) or retrograde (femoral with steerable introducer sheaths) approaches [10].

**Figure 2:**
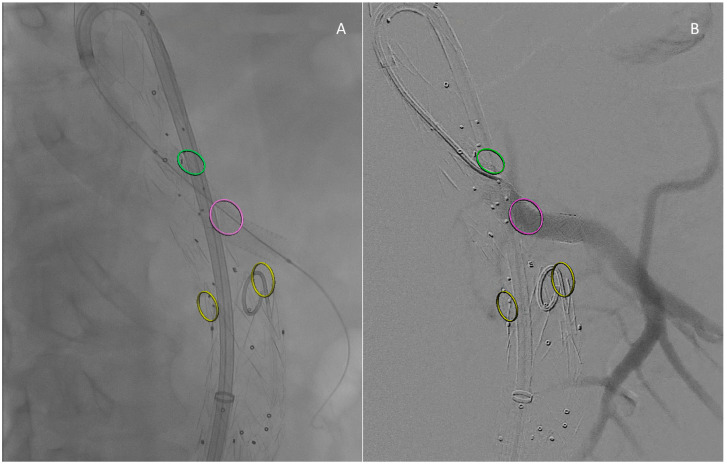
Intraoperative images: Result after bridging component deployment (**A**). Angiographic control (**B**). Circles mark the origin of reno–visceral vessels according to fusion guidance: green is for the Celiac Trunk, purple for the Superior Mesenteric Artery (the vessel just bridged), and yellow for the renal arteries.

In line with the standard technique, once an introducer sheath is positioned near the target branch, a 6 Fr Select Neuron catheter (125 cm in length, with H1 or BERN shape) is coupled with a 0.035″ hydrophilic guidewire to cannulate the branch and target vessel. The tapered catheter tip ensures atraumatic placement. The seamless transition zone allows for coaxial introducer sheath advancement within the branch but not inside the target vessel to avoid damage (Figure 1) [11]. The large 0.40″ inner lumen permits diagnostic angiography without over-the-wire exchange. Contrast injection confirms the catheter position and enables measuring the bridging component length with the 8 cm radiopaque tip aligned proximally with the branch inlet (Figure 1A). This eliminates the need for additional maneuvers or gap length measurements. The bridging component is therefore deployed and its patency is checked by injecting contrast media in the standard fashion (Figure 2).

The approach can be repeated for each target visceral vessel.

## 2. Discussion

Selecting the appropriate bridging component length is critical in complex endovascular treatments. However, the technique for sizing the bridge component is rarely discussed in the BEVAR literature, as surgeons often develop their own methods through practical experience. Common techniques include using a sizing catheter with radiopaque markers or a balloon catheter to estimate bridge component length before inserting and advancing it. Other, less precise approaches involve using the known length of the endograft’s branch as a reference to estimate the gap to be bridged, or utilizing pre-procedural axial length measurements, though the reliability may vary depending on the actual deployment height of the endograft. Compared to these methods, the proposed technique reduces the steps for each target vessel, saving time and reducing the risk of vessel loss or damage due to system instability. Moreover, the cost of the Neuron catheter is lower than combining a diagnostic catheter with a sizing or balloon catheter. An alternative inexpensive technique involves the operator holding the guiding catheter segment outside the introducer sheath valve between the thumb and index finger. Upon retracting the catheter, the bridging length corresponds to the distance between the operator’s fingers and the sheath’s hemostatic valve, which can be easily measured with a ruler. However, this method could be approximate and unsafe if the introducer sheath is not positioned within the target vessel due to guidewire displacement risk during pull-back maneuvers. While the Neuron catheter’s size could limit its use in small vessels or ostial stenosis cases, this is rare due to the tapered catheter tip, which also provides support advantages.

## 3. Conclusions

Standardizing a technique simplifies complex aortic procedures strategically and temporally. This described technique has been employed for years in the authors’ institution and presents a simple yet effective tool to mitigate the risk of vessel loss and injury, and reduce procedural and fluoroscopy time during complex aortic repair, saving at passage for each target visceral vessel overstenting. The decision to employ this catheter depends on the operators’ judgment.

Challenges may arise at any point in complex aortic procedures, making them both intimidating and stimulating. Operators must rely on their experience and skills to find solutions when hurdles occur.

## Data Availability

No new data were created or analyzed in this study.
